# Stress, Burnout, and Resilience among Healthcare Workers during the COVID-19 Emergency: The Role of Defense Mechanisms

**DOI:** 10.3390/ijerph18105258

**Published:** 2021-05-14

**Authors:** Mariagrazia Di Giuseppe, Gianni Nepa, Tracy A. Prout, Fabrizio Albertini, Stefano Marcelli, Graziella Orrù, Ciro Conversano

**Affiliations:** 1 Department of Surgical, Medical and Molecular Pathology, Critical and Care Medicine, University of Pisa, 56126 Pisa, Italy; graziella.orru@unipi.it (G.O.); ciro.conversano@unipi.it (C.C.); 2 Hospital “G. Mazzini”, ASL 4, 64100 Teramo, Italy; gianni.nepa@hotmail.it; 3 Ferkauf Graduate School of Psychology, Yeshiva University, Bronx, New York, NY 10461, USA; tracy.prout@yu.edu; 4 Hospital “G. Mazzoni”, Asur Marche Area Vasta 5 Ascoli Piceno, 63100 Ascoli Piceno, Italy; fabrizio.albertiniap@gmail.com (F.A.); s.marcelli@staff.univpm.it (S.M.); 5 Faculty of Medicine and Surgery, Marche Polytechnic University, 60131 Ancona, Italy

**Keywords:** COVID-19, stress, burnout, resilience, defense mechanisms, frontline workers, emotion regulation

## Abstract

The experience of working on the frontlines of the COVID-19 healthcare crisis has presented a cumulative traumatic experience that affects healthcare professionals’ well-being. Psychological resources such as resilience and adaptive defense mechanisms are essential in protecting individuals from severe stress and burnout. During September 2020, 233 healthcare workers responded to an online survey to test the impact of demographic variables, COVID-19 exposure, and psychological resources in determining stress and burnout during the COVID-19 emergency. Frontline workers reported higher scores for stress, emotional exhaustion, and depersonalization (*p* < 0.001) as compared to colleagues working in units not directly serving patients with COVID-19. Mature defensive functioning was associated with resilience and personal accomplishment (*r* = 0.320; *p* < 0.001), while neurotic and immature defenses were related to perceived stress and burnout. Stress and burnout were predicted by lower age, female gender, greater exposure to COVID-19, lower resilience, and immature defensive functioning among healthcare professionals (R^2^ = 463; *p* < 0.001). Working on the frontlines of the COVID-19 pandemic appears to provoke greater stress and burnout. On the other hand, resilience and adaptive defense mechanisms predicted better adjustment. Future reaction plans should promote effective programs offering support for healthcare workers who provide direct care to patients with COVID-19.

## 1. Introduction

One year after its emergence, the coronavirus disease 2019 (COVID-19) remains a global threat that impacts multiple aspects of human life. Lockdown and shelter-in-place measures have been employed to help alleviate the burden on healthcare systems worldwide. These measures have come at a high cost, resulting in job reductions, educational disruptions, and social restrictions [1] The reorganization of medical departments and health professional resources in order to prioritize care for COVID-19 patients has led to the strong reduction of quality of care and prevention of chronic diseases [2,3,4]. Moreover, the increased pressures on healthcare workers—including increased risk of infection and vicarious trauma among frontline healthcare workers—suggests the importance of monitoring and responding to the distress experienced by those on the frontlines of the ongoing pandemic [5,6,7].

The impact of pandemics on mental health has been well documented. Several studies from this and prior pandemics have demonstrated that socio-demographic characteristics and outbreak exposure are risk factors for the development of psychological distress among the general population [8,9,10,11,12]. In particular, specific factors—including younger age, female gender, longer lockdown conditions, and higher occurrence of positive cases among close relatives and friends—appear to predict worse symptoms of depression, anxiety, and post-traumatic stress [13,14,15,16,17]. Moreover, vulnerable groups, such as chronically ill patients, racial and ethnic minorities, and people with lower socio-economic status, have a higher risk of distress and worse psychological outcomes [18,19,20,21,22,23]. 

With regard to frontline health care workers, a number of studies have demonstrated that healthcare professionals have experienced higher levels of distress as compared to the general population, and, therefore, they are at high risk for the development of psychopathology [24,25,26,27,28]. Prevalence of insomnia, anxiety, depression, somatization, and obsessive-compulsive symptoms were higher in medical workers than in non-medical workers in China [29]. In a large sample of 1,257 health care professionals, Lai and colleagues [30] found that symptoms of depression, anxiety, insomnia, and distress were reported by 50.4%, 44.6%, 34.0%, and 71.5% of participants, respectively. Higher levels of psychological distress were found in nurses and technicians than in physicians, whereas burnout, defined as a syndrome of emotional exhaustion, cynicism, depersonalization, and perceived inefficacy resulting from long-term job stress that can occur among individuals who work with people in some capacity [31], was higher in doctors than in nurses and technicians [32,33,34]. Conversely, clear communication from the organization, social support, personal sense of control, and emotional regulation were found as protective factors against psychological distress in healthcare professionals [35,36,37,38]. However, there is still lack of empirical evidence demonstrating the increased risk of mental problems as consequence of the pandemic.

In addition to conscious coping mechanisms, individuals rely on unconscious operations known as defense mechanisms, which can mediate reactions to traumatic experiences and protect the individual from the awareness of feelings and thoughts of internal conflicts and external stressors [39,40,41]. An overview of the hierarchical organization of defense mechanism is displayed in Table 1.

Numerous studies have demonstrated that the maturity of defensive functioning is a protective factor against the development of psychological and somatic diseases [42,43,44,45]. Traumatic experiences can provoke a decrease in overall defensive functioning and increase the use of mental inhibition defense mechanisms, also called neurotic defenses. Individuals who have experience trauma tend to use defense mechanisms as repression, dissociation, and isolation of affects in order to protect themselves from the awareness of stressful experience that they cannot fully manage [46,47]; this allows them to maintain emotional distance from feeling or ideas associated with the stressor. High-adaptive defense mechanisms, also called mature defenses, moderate the individual’s adjustment to stressful conditions and foster resilience [48,49]. The function of mature defenses includes reduction of negative affect, partial or full awareness of stressful agents, and the ability to reflect and act upon the resolution of such conflicts. When mature or middle-adaptive defenses are ineffective or cannot be activated, the individual may begin to rely on less adaptive (e.g., immature) defenses [50]. The present study investigated the psychological impact of COVID-19 among healthcare workers and analyzed the role of defense mechanisms in offering protection from stress and burnout and in enhancing resilience and adjustment.

### Aims and Hypotheses

This study aimed to identify protective factors against perceived stress and burnout and factors that may enhance resilience among health workers. We analyzed socio-demographic characteristics, exposure to COVID-19, vicarious trauma, resilience, and defense mechanisms in order to test the following hypotheses: (1) healthcare professionals working directly with COVID-19 patients would demonstrate higher levels of stress and burnout and lower resilience than colleagues working in other units; (2) mature defensive functioning would be associated with lower stress and burnout and higher resilience; (3) inhibited/avoidant and immature defensive functioning would be associated with higher distress and lower resilience; and (4) sociodemographic variables, COVID-19 exposure, resilience, and defensive functioning would predict self-reported stress and burnout among healthcare professionals.

## 2. Methods

### 2.1. Participants and Procedures

Participants were 233 healthcare workers working in several Italian hospitals (47.7% completion rates). They were mostly female (*n* = 148; 62%) and 41 years of age on average (*M* = 41.49; *SD* = 10.38). Nurses represented 80% of the sample (*n* = 185), while the remaining 20% was equally distributed among physicians and healthcare aide workers. Twenty-two percent of the sample (*n* = 51) were frontline workers in COVID-19 units. Education higher that college degree was reported in 84% of the sample (*n* = 196), with 17% and 11% having master’s (*n* = 39) and doctoral degrees (*n* = 25), respectively. Participants’ working experience was 14.8 years on average (*SD* = 10.35), ranging from 0 to 40 years of experience in health care. Positive cases among personnel of the same unit were reported in less than 50% of the sample (*n* = 100; 43%), with 3% of participants (*n* = 7) reporting more than 15 colleagues infected with COVID-19. The rate of positive cases among patients was slightly higher. More than half of respondents (*n* = 134; 57%) reported the absence of positive cases among patients in their unit, whereas 10% of the sample (*n* = 25) reported more than 15 patients testing positive in their department.

The convenience sample was recruited among healthcare workers employed in several hospitals on Central Italy. The platform used for the survey was Google Form and the link was disseminated via text message and emails with up to three reminders for participant. Those who provided informed consent and completed an online survey investigating socio-demographics, professional status, exposure to COVID-19, and psychological variables were included in the study. Data were collected during September 2020. The study procedure was reviewed and approved by the local [omitted for peer review] Institutional Review Board.

### 2.2. Measures

Stress levels were assessed with the Perceived Stress Scale (PSS) [51], a 10-item questionnaire assessing the degree to which external demands exceed the individual’s perceived ability to cope. Items on the PSS are rated using a 5-point Likert scale, with total scores ranging from 0 to 40. The total score is calculated by summing item scores, with higher scores indicating higher perceived stress. Internal consistency in the present study was α = 0.87.

Burnout was measured with the Maslach Burnout Inventory (MBI) [52], a 22-item questionnaire designed for those working in healthcare and social service fields. Items on the MBI are rated on a 7-point Likert scale, which can then be used to calculate three subscales measuring emotional exhaustion, depersonalization, and personal accomplishment. The three scores are not combined into a global score and each subscale has distinct benchmarks (MBI-EE: low 0–16, moderate 17–26, high 27–54; MBI-D: low 0–6, moderate 7–12, high 13–35; MBI-PA: low 0–31, moderate 32–38, high 39–48). In the present study, internal consistency (Cronbach’s alpha) for the subscales was 0.92 (EE), 0.75 (D), and 0.82 (PA).

Resilience was assessed using the 14-item Resilience Scale (RS-14) [53], a 14-item questionnaire that uses a 7-point Likert scale to measure the ability to withstand or adaptively recover from stress. Total score ranges from 14 to 98, with higher scores indicating greater resilience. Internal consistency for the RS-14 has been reported as 0.90 in prior studies [53]; in the current study, Cronbach’s alpha was 0.90.

A wide range of defense mechanisms were assessed using the Defense Mechanisms Rating Scales-Self-Report-30 (DMRS-SR-30) [54]. The DMRS-SR-30 is a 30-item 5-point Likert scale questionnaire assessing the whole hierarchy of defense mechanisms. The DMRS-SR-30 provides scores for the overall defensive functioning (ODF), three defensive factors and seven defense levels, both hierarchically ordered based on their functions and level of adaptiveness. From least to most adaptive, DMRS-SR-30 factors are: immature-depressive defenses (acting out, passive aggression, help-rejecting complaining, splitting of self and others’ images, projective identification, projection, and devaluation), mental inhibition and avoidance defenses (denial, rationalization, autistic fantasy, idealization, and omnipotence), and mature defenses (affiliation, altruism, anticipation, humor, self-assertion, self-observation, sublimation, and suppression). Strong reliability and validity (e.g., criterion, concurrent, convergent, and discriminant) has been found for ODF and the defense factors, with moderate to high internal consistency for the defense levels subscales [54]. In the current study, Cronbach’s alpha for the ODF was 0.91; alphas for the three factors of the DMRS-SR-30 were 0.85 (immature-depressive), 0.83 (mental inhibition/avoidance), and 0.72 (mature). 

### 2.3. Statistical Analyses

Pearson correlations were used to examine associations between perceived stress, burnout, resilience, and defensive functioning. Independent samples *t*-tests were used to compare differences in perceived stress, burnout, and resilience between healthcare professionals working in COVID-19 units and their colleagues in other departments. Finally, hierarchical multiple regression was used to predict perceived stress and burnout by demographics, professional status, COVID-19 exposure, and defensive functioning.

## 3. Results

Independent samples *t*-tests were used to compare levels of stress, burnout, and resilience among healthcare workers with direct COVID-19 exposure with that of their counterparts working in other settings. Frontline COVID-19 healthcare workers reported significantly higher perceived stress and two aspects of burnout (e.g., emotional exhaustion and depersonalization) as compared to healthcare staff working in non- COVID-19 units. No differences emerged between these groups on the third component of burnout (personal accomplishment) or resilience, which were similarly reported in both frontline healthcare professionals and their colleagues working in other units. Table 2 presents results of these *t*-tests.

Pearson correlations were calculated to examine the second and third hypotheses—(2) that mature defenses would be associated with lower levels of stress and burnout and higher resilience and (3) that inhibited/avoidant and immature defensive functioning would be associated with greater stress and burnout, as well as lower resilience. These hypotheses were supported, with mature defenses inversely associated stress and burnout and positively associated with resilience and personal accomplishment. As expected, neurotic and immature defenses followed the opposite trend. Both of these defense factors showed positive correlations with stress and burnout (ranging from *r* = 0.129 to *r* = 0.377), and negatively correlated with resilience and personal accomplishment (ranging from *r* = −0.190 to *r* = −0.322). Immature defenses were most strongly associated with perceived stress and emotional exhaustion, whereas neurotic defenses demonstrated the strongest correlation with depersonalization. In addition, the maturity of defensive functioning, expressed as ODF, was associated with personal accomplishment (*r* = 0.305; *p* < 0.001) and resilience (*r* = 0.282; *p* < 0.001), whereas lower adaptive ODF was associated with perceived stress, emotional exhaustion, and depersonalization (ranging from *r* = −0.209 to *r* = −0.477; all *p* values < 0.001). Full results can be seen in Table 3.

A series of hierarchical multiple regressions were run to test predictors of perceived stress and two burnout indexes (emotional exhaustion and depersonalization). In each regression, demographic variables of age and gender were entered in the first model, followed by COVID-19 exposure, resilience, and defensive functioning in the second, third, and fourth models, respectively.

Table 4 shows results from a hierarchical multiple regression analysis performed with perceived stress as the dependent variable. Age and gender combined explained 19.6% of the variance (*p* < 0.0001), with age accounting for three-fourths of the total. COVID-19 exposure accounted for 6.3% (*p* < 0.0001) of the variance, while resilience explained an additional 9.4% (*p*<.0001). The inclusion of the defensive functioning scales significantly increased the variance by 11.1%, with total variance explained by the model totaling 46.3% (*F* = 39.117, *p* < 0.0001). In this final model (Model 5), lower age, female gender, higher exposure to COVID-19, lower resilience, and less adaptive defensive functioning were the best predictors of perceived stress among healthcare professionals.

Hierarchical multiple regression analysis was performed with emotional exhaustion as the dependent variable (see Table 5). Demographics included in the first model explained only 2.7% of the variance (*p* = 0.011). The addition of COVID-19 exposure increased the variance explained by 9.4% (*p* < 0.0001). Modest increases of explained variance were found when resilience and defense mechanisms were added to the model, each accounting for about 2% of the variance. The final model (Model 4) explained 16.4% of the total variance (*F* = 11.150, *p* < 0.0001) and indicated that lower age, higher exposure to COVID-19, lower resilience, and less adaptive defensive functioning were the best predictors of emotional exhaustion in healthcare professionals.

To identify predictors of depersonalization, a final hierarchical multiple regression analysis was conducted. Age and gender explained 5% and 2.3% of the variance, respectively. The inclusion of COVID-19 exposure significantly increased the explained variance by 9.8% (*p* < 0.0001), whereas only a modest increase was found including defensive functioning in the model. In the final model (Model 4, see Table 6), lower age, female gender, higher exposure to COVID-19, and lower defensive functioning were the best predictors of depersonalization, accounting for 19.5% of the explained variance (*F* = 13.815, *p* < 0.0001).

## 4. Discussion

Working on the frontlines of the COVID-19 pandemic has been defined as a traumatic experience that affects healthcare professionals’ well-being [55,56]. Uncertainty about COVID-19 safety and contagion, direct exposure to patient suffering and death, fear of getting infected or spreading the infection among colleagues and relatives, physical exhaustion due to the overwhelming workload, and concerns about institutional management of the pandemic are just some of the reasons for the increased risk of stress and burnout among frontline healthcare workers [57,58]. Defense mechanisms are psychological emotion regulation strategies that foster resilience and, thus, better adjustment to the stressful experience of the COVID-19 pandemic [59]. The present study provides empirical evidence of the impact of the COVID-19 outbreak on the psychological functioning of healthcare workers and of the role of psychological resources in enhancing better adjustment. Findings highlighted that the maturity of defensive functioning is associated to healthcare workers’ psychological well-being and their fails are related to greater distress and lower resilience. 

This study provides evidence of the unique burden placed upon healthcare workers providing care on the frontlines of the COVID-19 pandemic. Findings confirmed that psychological distress was higher in in medical staff working in COVID-19 units as compared to colleagues working in other healthcare departments. Frontline workers reported higher level of perceived stress, emotional exhaustion and depersonalization than their colleagues working in other units. It may be, as some have argued, that direct work with COVID-19 patients constitutes a cumulative vicarious trauma for frontline workers with direct effects on stress and burnout [60,61]. Notably, no differences were found in personal accomplishment and resilience among these two groups of healthcare workers, perhaps suggesting that the structural components of strengths-based, psychological functioning are not influenced by time-limited stressful experiences.

The association of defensive functioning with perceived stress, burnout, and resilience was also confirmed by our findings [62]. Higher overall defensive functioning and higher use of mature defenses were related to a greater sense of personal accomplishment and ability to withstand or adaptively recover from stress and, conversely, to lower perceived stress, emotional exhaustion and depersonalization. Accordingly, the use of neurotic and immature defenses was associated with greater stress and burnout and lower resilience, suggesting the possibility that implicit emotion regulation (e.g., defense mechanisms) may help protect the individual from internal and external stressors. In particular, mental inhibition and avoidance defenses, which include defenses that keep either the emotional or the cognitive components of a conflict out of the individual’s awareness (i.e., repression, dissociation, isolation of affects) showed a greater relationship with depersonalization. Immature-depressive defenses, which include the least adaptive defenses in the hierarchy (i.e., passive aggression, hypochondriasis, splitting of self and other’s image) were associated with emotional exhaustion. These findings highlight the inter-relationship between emotion regulation and adjustment and suggest the importance of studying the relationship between specific defense mechanisms and reported psychological symptoms [63,64,65].

Interesting findings emerged from hierarchical multiple regression analyses run to identify the best predictors of perceived stress and burnout among this sample of healthcare workers. We expected that higher exposure to COVID-19 and lower maturity of defense mechanisms would predict distress in healthcare professionals. Overall, results confirmed this hypothesis, highlighting differences between the two constructs of stress and burnout. Younger age and defensive functioning were the strongest predictors of perceived stress, while COVID-19 exposure was identified as the best predictor of both burnout symptoms of emotional exhaustion and depersonalization. In other words, findings revealed that perceived stress is influenced by an overall psychological immaturity that affect the individual’s ability to cope with internal conflict and stressful life events, whereas burnout is highly affected by direct, intense, and prolonged exposure to chronic interpersonal stressors on the job [66]. Further research should be designed for analyzing effective protective factors for the prevention of burnout among healthcare workers. The overwhelming workload experience by healthcare workers over the past year of the COVID-19 pandemic has presumably increased their risk of developing the burnout syndrome. Moreover, the employment of additional younger, early career, medical staff working in critical conditions, could increase the risk of stress among poorly psychologically equipped frontline healthcare workers [67]. 

### Limitations and Future Directions

The present study has several limitations. The small sample makes it difficult to generalize results to the broader population of healthcare workers. Further investigations in larger case-control samples should be done to confirm these findings. Moreover, the length of the survey, administered in a period in which the workload was overwhelming, limited the number of participants. Shorter surveys might increase the sample size, although at the expense of data complexity. Furthermore, the cross-sectional research design did not allow inferences about the causal relations among studied variables and limited our speculation to associations between different aspects of psychological functioning. Prospective longitudinal studies might inform on causal long-term effects of COVID-19 frontline work on psychological functioning. Finally, the use of self-reported measures is subject to demand characteristics and bias. Future studies should consider the implementation of structured interviews coded by expert raters for the assessment of implicit emotion regulation. Further investigations should be done to test whether the duration and the severity of this traumatic exposure may impact stable aspects of personality structure.

## 5. Conclusions

The present study demonstrated the impact of COVID-19 in healthcare workers’ psychological well-being and pointed out the key role of defense mechanisms as protective factor against stress and burnout. As the COVID-19 pandemic continues, it is important to prevent and mitigate psychological distress among healthcare professionals [68,69,70]. While the global vaccine campaign has begun, the prevention and treatment of psychological distress for frontline workers has not been adequately addressed [71]. Future response plans should include adequate psychosocial support, counseling, stress management programs, telemedicine and informal support groups for healthcare workers who are one of our most valuable resources in the fight against the COVID-19 pandemic [72,73]

## Figures and Tables

**Table 1:** DMRS-SR-30 quantitative scoring system.

Defensive Category	Defense Level	Defense Mechanism
Mature	High adaptive	affiliation
		altruism
		anticipation
		humor
		self-assertion
		self-observation
		sublimation
		suppression
Neurotic ^a^	Obsessional	intellectualization
		isolation of affect
		undoing
	Neurotic ^b^	displacement
		dissociation
		reaction formation
		repression
Immature ^c^	Minor image-distorting	devaluation
		idealization
		omnipotence
	Disavowal	denial
		projection
		rationalization
		autistic fantasy
	Major image-distorting	projective identification
		splitting of self-image
		splitting of other’s image
	Action	acting out
		help-rejecting complaining
		passive aggression

^a^ The Neurotic category includes all defense mechanisms belonging to obsessional and neurotic defense levels. ^b^ The Neurotic defense level includes two sublevels of Hysterical (including repression and dissociation) and Other Neurotic (including reaction formation and displacement) defense levels. ^c^ The Immature category includes two categories of Depressive and Other immature (or non-depressive) defenses. The Depressive category includes all Action and Major image-distorting defenses, plus projection and devaluation. Other immature category includes autistic fantasy, rationalization, denial, omnipotence, and idealization.

**Table 2:** Differences in stress, burnout, and resilience between COVID-19 frontline workers (*N* = 51) and healthcare professionals working in other units (*N* = 182).

	COVID-19 Frontline Workers		Other Healthcare Professionals			
	*Mean*	*SD*	*Mean*	*SD*	*t*	*p*
Perceived Stress	21.43	6.02	16.27	6.22	−5.270	<0.001
Emotional Exhaustion	28.41	14.27	17.75	12.21	−5.306	<0.001
Depersonalization	9.98	8.15	4.95	5.09	−5.387	<0.001
Personal Accomplishment	36.31	9.11	37.78	9.15	1.008	0.314
Resilience	73.08	12.93	75.73	11.38	1.426	0.155

Note: cut-off points for MBI subscales. Emotional exhaustion: low 0–16, moderate 17–26, high 27–54; depersonalization: low 0–6, moderate 7–12, high 13–35; personal accomplishment: low 0–31, moderate 32–38, high 39–48.

**Table 3:** Pearson correlations between psychological variables tested in health care professionals at the time of COVID-19 (*N* = 233).

	Defense Mechanisms Rating Scale-Self Report-30 (DMRS-SR-30)			
	ODF	Factor 1	Factor 2	Factor 3
		Mature Defenses	Mental Inhibition/Avoidance Defenses	Immature/Depressive Defenses
Perceived Stress	−0.477 **	−0.484 **	0.354 **	0.377 **
Emotional Exhaustion	−0.245 **	−0.240 **	0.129 *	0.218 **
Depersonalization	−0.209 **	−0.257 **	0.221 **	0.177 **
Personal Accomplishment	0.305 **	0.320 **	−222 **	−0.257 **
Resilience	0.282 **	0.321 **	−0.322 **	−0.190 **

* *p* < 0.01; ** *p* < 0.001.

**Table 4:** Hierarchical regression analysis of predictors of perceived stress (*N* = 233).

	b’	t	*p*	F	R^2^	Changed R^2^	*p*
Model 1				42.050	0.154	0.154	<0.0001
Age	−0.392	−6.485	<0.0001				
Model 2				27.952	0.196	0.042	<0.0001
Age	−0.385	−6.497	<0.0001				
Gender	0.204	3.446	0.001				
Model 3				26.581	0.258	0.063	<0.0001
Age	−0.342	−5.915	<0.0001				
Gender	0.286	3.254	0.001				
COVID-19	0.255	4.402	<0.0001				
Model 4				30.968	0.352	0.094	<0.0001
Age	−0.320	−5.893	<0.0001				
Gender	0.179	3.344	0.001				
COVID-19	0.230	4.234	<0.0001				
Resilience	−0.308	−5.744	<0.0001				
Model 5				39.117	0.463	0.111	<0.0001
Age	−0.299	−6.034	<0.0001				
Gender	0.180	3.724	<0.0001				
COVID-19	0.168	3.325	0.001				
Resilience	−0.215	−4.235	<0.0001				
ODF	−0.354	−6.843	<0.0001				

Notes: Hierarchical multiple regression analysis with dependent variable the perceived stress scale. In this analysis, age and gender were entered in the first block, COVID-19 exposure was added in the second block, resilience was added in the third block, and overall defensive functioning was added in the fourth block.

**Table 5:** Hierarchical regression analysis of predictors of emotional exhaustion (*N* = 233).

	b’	t	*p*	F	R^2^	Changed R^2^	*p*
Model 1				6.503	0.027	0.027	0.011
Age	−0.165	−2.550	0.011				
Model 2				15.822	0.121	0.094	<0.0001
Age	−0.113	−1.794	0.074				
COVID-19	0.301	4.948	<0.0001				
Model 3				12.920	0.145	0.024	<0.0001
Age	−0.101	−1.630	0.105				
COVID-19	0.298	4.786	<0.0001				
Resilience	−0.155	−2.525	0.012				
Model 4				11.150	0.164	0.019	<0.0001
Age	−0.093	−1.508	0.133				
COVID-19	0.273	4.351	<0.0001				
Resilience	−0.117	−1.853	0.065				
ODF	−0.146	−2.267	0.024				

Notes: Hierarchical multiple regression analysis with dependent variable the MBI emotional exhaustion scale. In this analysis, age and gender were entered in the first block, COVID-19 exposure was added in the second block, resilience was added in the third block, and overall defensive functioning was added in the fourth block.

**Table 6:** Hierarchical regression analysis of predictors of depersonalization (*N* = 233).

	b’	t	*p*	F	R^2^	Changed R^2^	*p*
Model 1				12.036	0.050	0.050	0.001
Age	−0.223	−3.469	0.001				
Model 2				8.982	0.072	0.023	<0.0001
Age	−0.228	−3.594	<0.0001				
Gender	−0.152	−2.384	0.018				
Model 3				15.629	0.170	0.098	<0.0001
Age	−0.175	−2.865	0.005				
Gender	−0.174	−2.883	0.004				
COVID-19	0.318	5.187	<0.0001				
Model 4				13.815	0.195	0.025	<0.0001
Age	−0.152	−2.496	0.013				
Gender	−0.162	−2.707	0.007				
COVID-19	0.287	4.671	<0.0001				
ODF	−0.164	−2.668	0.008				

Notes: Hierarchical multiple regression analysis with dependent variable the MBI depersonalization scale. In this analysis, age and gender were entered in the first block, COVID-19 exposure was added in the second block, resilience was added in the third block, and overall defensive functioning was added in the fourth block.

## Data Availability

The data presented in this study are available on request from the corresponding author. The data are not publicly available due to ethical restrictions.

## References

[1] Nicola M., Alsafi Z., Sohrabi C., Kerwan A., Al-Jabir A., Iosifidis C., Agha M., Agha R. (2020). The socio-economic implications of the coronavirus pandemic (COVID-19): A review. Int. J. Surg..

[2] Békés V., Aafjes-van Doorn K., Prout T.A., Hoffman L. (2020). Stretching the analytic frame: Analytic therapists’ experiences with remote therapy during COVID-19. J. Am. Psychoanal. Assoc..

[3] Dieset I., Løvhaug L., Selle M., Kolseth A., Smeland O.B., Færden A. (2021). Lessons learned from a cross-sectional survey among patients and staff in an acute psychiatric unit during an ongoing pandemic outbreak. Psychiatry Res..

[4] Sardella A., Lenzo V., Bonanno G.A., Martino G., Basile G., Quattropani M.C. (2021). Dispositional optimism and context sensitivity: Psychological contributors to frailty status among elderly outpatients. Front. Psychol..

[5] Lenzo V., Quattropani M.C., Sardella A., Martino G., Bonanno G.A. (2021). Depression, anxiety, and stress among healthcare workers during the COVID-19 outbreak and relationships with expressive flexibility and context sensitivity. Front. Psychol..

[6] Nguyen L.H., Drew D.A., Graham M.S., Joshi A.D., Guo C.-G., Ma W., Mehta R.S., Warner E.T., Sikavi D.R., Lo C.-H. (2020). Risk of COVID-19 among frontline healthcare workers and the general community: A prospective cohort study. Lancet Public Health.

[7] Merlo E.M., Stoian A., Motofei I.G., Settineri S. (2020). Clinical psychological figures in healthcare professionals: Resilience and maladjustment as the “cost of care”. Front. Psychol..

[8] Brailovskaia J., Cosci F., Mansueto G., Miragall M., Herrero R., Baños R.M., Krasavtseva Y., Kochetkov Y., Margraf J. (2021). The association between depression symptoms, psychological burden caused by Covid-19 and physical activity: An investigation in Germany, Italy, Russia, and Spain. Psychiatry Res..

[9] Castellano-Tejedor C., Torres-Serrano M., Cencerrado A. (2021). Psychological impact in the time of COVID-19: A cross-sectional population survey study during confinement. J. Health Psychol..

[10] Charles N.E., Strong S.J., Burns L.C., Bullerjahn M.R., Serafine K.M. (2021). Increased mood disorder symptoms, perceived stress, and alcohol use among college students during the COVID-19 pandemic. Psychiatry Res..

[11] Orrù G., Bertelloni D., Diolaiuti F., Mucci F., Di Giuseppe M., Biella M., Gemignani A., Ciacchini R., Conversano C. (2021). Long-COVID Syndrome? A study on the persistence of neurological, psychological and physiological symptoms. Healthcare.

[12] Settineri S., Merlo E.M. (2020). Editorial: Fear of contamination. Mediterr. J. Clin. Psychol..

[13] Conversano C., Di Giuseppe M., Miccoli M., Ciacchini R., Gemignani A., Orrù G. (2020). Mindfulness, age and gender as protective factors against psychological distress during COVID-19 pandemic. Front. Psychol..

[14] Daly Z., Slemon A., Richardson C.G., Salway T., McAuliffe C., Gadermann A.M., Thomson K.C., Hirani S., Jenkins E.K. (2021). Associations between periods of COVID-19 quarantine and mental health in Canada. Psychiatry Res..

[15] Mazza C., Orrù G., Burla F., Monaro M., Ferracuti S., Colasanti M., Roma P. (2019). Indicators to distinguish symptom accentuators from symptom producers in individuals with a diagnosed adjustment disorder: A pilot study on inconsistency subtypes using SIMS and MMPI-2-RF. PLoS ONE.

[16] Rossell S.L., Neill E., Phillipou A., Tan E.J., Toh W.L., Van Rheenen T.E., Meyer D. (2021). An overview of current mental health in the general population of Australia during the COVID-19 pandemic: Results from the COLLATE project. Psychiatry Res..

[17] Settineri S., Merlo E.M., Frisone F., Marchetti D., Verrocchio M.C., Pellegrino M.G., Mento C., Fenga C. (2018). The experience of health and suffering in the medical profession. Mediterr. J. Clin. Psychol..

[18] Catalano A., Martino G., Bellone F., Papalia M., Lasco C., Basile G., Sardella A., Nicocia G., Morabito N., Lasco A. (2019). Neuropsychological assessment in elderly men with benign prostatic hyperplasia treated with dutasteride. Clin. Drug Investig..

[19] Martino G., Catalano A., Agostino R.M., Bellone F., Morabito N., Lasco C.G., Vicario C.M., Schwarz P., Feldt-Rasmussen U. (2020). Quality of life and psychological functioning in postmenopausal women undergoing aromatase inhibitor treatment for early breast cancer. PLoS ONE.

[20] Martino G., Caputo A., Vicario C.M., Feldt-Rasmussen U., Watt T., Quattropani M.C., Benvenga S., Vita R. (2021). Alexithymia, emotional distress and perceived quality of life in patients with Hashimoto’s Thyroiditis. Front. Psychol..

[21] Orru G., Sampietro S., Catanzaro S., Girardi A., Najjar M., Giantin V., Sergi G., Manzato E., Enzi G., Inelmen E.M. (2009). Serial position effect in a free recall task: Differences between probable dementia of Alzheimer type (PDAT), vascular (VaD) and mixed etiology dementia (MED). Arch. Gerontol. Geriatr..

[22] Poudel K., Subedi P. (2020). Impact of COVID-19 pandemic on socioeconomic and mental health aspects in Nepal. Int. J. Soc. Psychiatry.

[23] Tai D., Shah A., Doubeni C.A., Sia I.G., Wieland M.L. (2020). The disproportionate impact of COVID-19 on racial and ethnic minorities in the United States. Clin. Infect. Dis..

[24] Conversano C., Ciacchini R., Orrù G., Di Giuseppe M., Gemignani A., Poli A. (2020). Mindfulness, compassion, and self-compassion among health care professionals: What’s new? A systematic review. Front. Psychol..

[25] Iasevoli M., Giantin V., Voci A., Valentini E., Zurlo A., Maggi S., Siviero P., Orrù G., Crepaldi G., Pegoraro R. (2012). Discussing end-of-life care issues with terminally ill patients and their relatives: Comparisons among physicians, nurses and psychologists. Aging Clin. Exp. Res..

[26] Ng Q.X., De Deyn M., Lim D.Y., Chan H.W., Yeo W.S. (2020). The wounded healer: A narrative review of the mental health effects of the COVID-19 pandemic on healthcare workers. Asian J. Psychiatry.

[27] Nochaiwong S., Ruengorn C., Awiphan R., Ruanta Y., Boonchieng W., Nanta S., Wongpakaran T. (2020). Mental health circumstances among health care workers and general public under the pandemic situation of COVID-19 (HOME-COVID-19). Medicine.

[28] Quattropani M.C., Lenzo V., Baio M., Bordino V., Germanà A., Grasso D., Pennica S. (2017). Metacognitive beliefs and coping strategies in homecare professionals at risk of burnout. Psicol. Della Salut..

[29] Zhang W.R., Wang K., Yin L., Zhao W.F., Xue Q., Peng M., Min B., Tian Q., Leng H., Du J. (2020). Mental health and psychosocial problems of medical health workers during the COVID-19 epidemic in China. Psychother. Psychosom..

[30] Lai J., Ma S., Wang Y., Cai Z., Hu J., Wei N., Wu J., Du H., Chen T., Li R. (2020). Factors associated with mental health outcomes among health care workers exposed to Coronavirus Disease 2019. JAMA.

[31] Maslach C., Jackson S.E., Leiter M.P. (1996). MBI: The Maslach Burnout Inventory Manual.

[32] Dosil M., Ozamiz-Etxebarria N., Redondo I., Picaza M., Jaureguizar J. (2020). Psychological symptoms in health professionals in Spain after the first wave of the COVID-19 pandemic. Front. Psychol..

[33] Khasne R.W., Dhakulkar B.S., Mahajan H.C., Kulkarni A.P. (2020). Burnout among Healthcare Workers during COVID-19 Pandemic in India: Results of a Questionnaire-Based Survey. Indian J. Crit. Care Med. Peer Rev. Off. Publ. Indian Soc. Crit. Care Med..

[34] Tan B., Kanneganti A., Lim L., Tan M., Chua Y.X., Tan L., Sia C.H., Denning M., Goh E.T., Purkayastha S. (2020). Burnout and associated factors among health care workers in Singapore during the COVID-19 pandemic. J. Am. Med. Dir. Assoc..

[35] De Brier N., Stroobants S., Vandekerckhove P., De Buck E. (2020). Factors affecting mental health of health care workers during coronavirus disease outbreaks (SARS, MERS & COVID-19): A rapid systematic review. PLoS ONE.

[36] Di Giuseppe M., Ciacchini R., Piarulli A., Nepa G., Conversano C. (2019). Mindfulness disposition and defense style as positive responses to psychology distress in oncology professionals. Eur. J. Oncol. Nurs..

[37] Jose S., Dhandapani M., Cyriac M.C. (2020). Burnout and resilience among frontline nurses during COVID-19 pandemic: A cross-sectional study in the emergency department of a tertiary care center, North India. Indian J. Crit. Care Med. Peer Rev. Off. Publ. Indian Soc. Crit. Care Med..

[38] Lenzo V., Bordino V., Bonanno G.A., Quattropani M.C. (2020). Understanding the role of regulatory flexibility and context sensitivity in preventing burnout in a palliative home care team. PLoS ONE.

[39] American Psychiatric Association (2013). Diagnostic and Statistical Manual of Mental Disorders.

[40] Perry J.C. (1990). Defense Mechanism Rating Scales (DMRS).

[41] Vaillant G.E. (1992). Ego Mechanisms of Defense: A Guide for Clinicians and Researchers.

[42] Conversano C., Di Giuseppe M. (2021). Psychological factors as determinants of chronic conditions: Clinical and psychodynamic advances. Front. Psychol..

[43] De Roten Y., Djillali S., Crettaz von Roten F., Despland J.N., Ambresin G. (2021). Defense mechanisms and treatment response in depressed inpatients. Front. Psychol..

[44] Martino G., Caputo A., Vicario C.M., Catalano A., Schwarz P., Quattropani M.C. (2020). The Relationship Between Alexithymia and Type 2 Diabetes: A Systematic Review. Front. Psychol..

[45] Martino G., Caputo A., Schwarz P., Bellone F., Fries W., Quattropani M.C., Vicario C.M. (2020). Alexithymia and inflammatory bowel disease: A systematic review. Front. Psychol..

[46] Hayden M.C., Müllauer P.K., Beyer K., Gaugeler R., Senft B., Dehoust M.C., Andreas S. (2021). Increasing mentalization to reduce maladaptive defense in patients with mental disorders. Front. Psychiatry.

[47] Perry J.C., Sigal J.J., Boucher S., Paré N., Ouimet M.C., Normand J., Henry M. (2005). Personal strengths and traumatic experiences among institutionalized children given up at birth: II: Adaptation in late adulthood. J. Nerv. Ment. Dis..

[48] Hersoug A.G., Wærsted M., Lau B. (2021). Defensive functioning moderates the effects of nondirective meditation. Front. Psychol..

[49] Tanzilli A., Di Giuseppe M., Giovanardi G., Boldrini T., Caviglia G., Conversano C., Lingiardi V. (2021). Mentalization, attachment, and defense mechanisms: A psychodynamic diagnostic manual-2-oriented empirical investigation. Res. Psychother. Psychopathol. Process Outcome.

[50] Di Giuseppe M., Perry J.C., Conversano C., Gelo O.C.G., Gennaro A. (2020). Defense mechanisms, gender, and adaptiveness in emerging personality disorders in adolescent outpatients. J. Nerv. Ment. Dis..

[51] Cohen S., Kamarck T., Mermelstein R. (1983). A global measure of perceived stress. J. Health Soc. Behav..

[52] Maslach C., Jackson S.E. (1981). The measurement of experienced burnout. J. Organ. Behav..

[53] Wagnild G. (2009). A review of the resilience scale. J. Nurs. Meas..

[54] Di Giuseppe M., Perry J.C., Lucchesi M., Michelini M., Vitiello S., Piantanida A., Fabiani M., Maffei S., Conversano C. (2020). Preliminary reliability and validity of the DMRS-SR-30, a novel self-report based on the defense mechanisms rating scales. Front. Psychiatry.

[55] Elbay R.Y., Kurtulmuş A., Arpacıoğlu S., Karadere E. (2020). Depression, anxiety, stress levels of physicians and associated factors in Covid-19 pandemics. Psychiatry Res..

[56] Selvaraj S., Reddy P.V., Muralidharan K., Gangadhar B.N. (2020). Impact of COVID-19 on mental health: A watershed moment in tertiary care service provision in India?. Asian J. Psychiatry.

[57] Chen Y., Zhou H., Zhou Y., Zhou F. (2020). Prevalence of self-reported depression and anxiety among pediatric medical staff members during the COVID-19 outbreak in Guiyang, China. Psychiatry Res..

[58] Tam C.W., Pang E.P., Lam L.C., Chiu H.F. (2004). Severe acute respiratory syndrome (SARS) in Hong Kong in 2003: Stress and psychological impact among frontline healthcare workers. Psychol. Med..

[59] Albott C.S., Wozniak J.R., McGlinch B.P., Wall M.H., Gold B.S., Vinogradov S. (2020). Battle buddies: Rapid deployment of a psychological resilience intervention for health care workers during the COVID-19 pandemic. Anesth. Analg..

[60] Lu W., Wang H., Lin Y., Li L. (2020). Psychological status of medical workforce during the COVID-19 pandemic: A cross-sectional study. Psychiatry Res..

[61] Orrù G., Marzetti F., Conversano C., Vagheggini G., Miccoli M., Ciacchini R., Panait E., Gemignani A. (2021). Secondary traumatic stress and burnout in healthcare workers during COVID-19 outbreak. Int. J. Environ. Res. Public Health.

[62] Walker G., McCabe T. (2021). Psychological defence mechanisms during the COVID-19 pandemic: A case series. Eur. J. Psychiatry.

[63] Di Giuseppe M., Zilcha-Mano S., Prout T.A., Perry J.C., Orrù G., Conversano C. (2020). Psychological impact of coronavirus disease 2019 among Italians during the first week of lockdown. Front. Psychiatry.

[64] Merlo E.M., Stoian A., Motofei I.G., Settineri S. (2021). The role of suppression and the maintenance of euthymia in the clinical settings. Front. Psychol..

[65] Prout T.A., Zilcha-Mano S., Aafjes-van Doorn K., Békés V., Christman-Cohen I., Whistler K., Kui T., Di Giuseppe M. (2020). Identifying predictors of psychological distress during COVID-19: A machine learning approach. Front. Psychol..

[66] Maslach C., Leiter M.P. (2016). Understanding the burnout experience: Recent research and its implications for psychiatry. World Psychiatry.

[67] Conti C., Fontanesi L., Lanzara R., Rosa I., Porcelli P. (2020). Fragile heroes. The psychological impact of the COVID-19 pandemic on health-care workers in Italy. PLoS ONE.

[68] Aafjes-Van Doorn K., Békés V., Prout T.A., Hoffman L. (2020). Psychotherapists’ vicarious traumatization during the COVID-19 pandemic. Psychol. Trauma Theory Res. Pract. Policy.

[69] Elkholy H., Tawfik F., Ibrahim I., Salah El-Din W., Sabry M., Mohammed S., Hamza M., Alaa M., Fawzy A.Z., Ashmawy R. (2020). Mental health of frontline healthcare workers exposed to COVID-19 in Egypt: A call for action. Int. J. Soc. Psychiatry.

[70] Shanafelt T., Ripp J., Trockel M. (2020). Understanding and addressing sources of anxiety among health care professionals during the COVID-19 pandemic. JAMA.

[71] Bao Y., Sun Y., Meng S., Shi J., Lu L. (2020). 2019-nCoV epidemic: Address mental health care to empower society. Lancet.

[72] Krystal J.H., McNeil R.L. (2020). Responding to the hidden pandemic for healthcare workers: Stress. Nat. Med..

[73] Ng Q.X., Chee K.T., De Deyn M., Chua Z. (2020). Staying connected during the COVID-19 pandemic. Int. J. Soc. Psychiatry.

